# Practice-Based Quality Improvement Initiative to Support Voluntary Frailty Prevention Through Autonomy-Supportive Dialogue

**DOI:** 10.7759/cureus.109010

**Published:** 2026-05-17

**Authors:** Masaki Yoneta, Tatehisa Seki, Dai Saiki

**Affiliations:** 1 Department of Home Care, Social Welfare Cooperation Kitano-Aikoukai, Kitami, JPN; 2 Medical and Care Coordination Support Center of Kitami City, Hokusei Memorial Hospital, Koshokai Medical Corporation, Kitami, JPN; 3 Center for the Strategy of Emergence, Japan Research Institute, Limited, Tokyo, JPN

**Keywords:** autonomy-supportive dialogue, frailty prevention in daycare settings, japanese geriatrics, person-centered long-term care, quality improvement in geriatric care

## Abstract

Background

Japan is experiencing rapid population aging, accompanied by increasing long-term care demand and workforce constraints, particularly in rural and semi-rural regions where rehabilitation professionals are scarce. Many daycare facilities continue to operate within a traditional service culture focused on routine caregiving rather than autonomy-supportive, preventive engagement. To address this practice gap, a quality improvement (QI) initiative introduced a structured dialogical approach designed to support intrinsic motivation and voluntary daily activity among older adults attending a daycare center.

Methods

This project was conducted as a practice-based QI initiative embedded within routine service delivery at a community daycare (day service) center in Kitami City, Hokkaido, Japan. The initiative was embedded in routine service delivery and consisted of semi-structured dialogues and follow-up conversations at each daycare visit that emphasized value clarification, reflective goal-setting, and self-directed activity planning. Participants who were not receiving external rehabilitation services and who engaged in at least one full dialogue cycle were included. QI outcomes were examined through (1) practice-based observations focusing on intervention adaptation and behavioral change and (2) exploratory quantitative assessment using the Short-Form Berg Balance Scale (SF-BBS) at baseline, six months, and 12 months.

Results

Thirty-seven users met eligibility criteria; 34 completed the six-month and 14 completed the 12-month follow-up. While guided by a common dialogical framework, practice-based adaptations emerged during implementation, particularly for participants with cognitive impairment, where repetition and simplified reflection appeared to support value recall and engagement. Descriptive observations suggested increased engagement in self-directed activity and voluntary participation in home-based exercise. Non-planned practice-based observations included enhanced peer interaction, autonomous use of the training area, and increased user-initiated requests for functional training. Exploratory SF-BBS analyses suggested a positive functional trend among participants with available follow-up data, within the limitations of a non-controlled QI context.

Conclusions

This QI initiative suggests that autonomy-supportive, dialogical engagement may represent a feasible practice-based modification of routine daycare interactions even within daycare facilities characterized by traditionally structured caregiving practices. The findings underscore the importance of relational dialogue, contextual adaptation, and practice-based learning in real-world implementation. Such an approach may offer transferable insights for geriatric and preventive care in aging societies, although further replication across multiple settings and with more comprehensive outcome assessment is warranted.

## Introduction

Japan is experiencing rapid population aging, accompanied by increasing long-term care demand and workforce constraints, particularly in rural and semi-rural regions. This demographic shift has led to a severe shortage of caregiving personnel and increasing fiscal pressure on local governments, particularly in rural regions [1,2]. Under these constraints, preventing long-term frailty among older adults has become an urgent policy priority. Daycare centers serve as key community resources for maintaining physical function and social participation; however, exercise opportunities in these settings are typically limited to one or two sessions per week and often lack sufficient duration or intensity to produce measurable physical gains [3,4].

In addition to these structural limitations, the prevailing service model, bringing older adults to the center and having them exercise, may unintentionally narrow self-determination and intrinsic motivation. Such externally regulated participation can hinder the autonomy necessary for sustained engagement in meaningful daily activities [5]. This challenge raises the possibility that approaches supporting internal motivation and voluntary activity may complement provider-driven exercise instruction in routine daycare practice.

Self-determination theory (SDT) provides a well-established framework for understanding and facilitating autonomous motivation in health behavior change [6]. Guided by SDT, this project sought to promote self-driven activity by fostering dialogical engagement between professionals and participants. The practical design also drew on Biestek’s principles of social work, including individualization, purposeful expression of feelings, acceptance, and self-determination [7]. In this initiative, these principles were translated into dialogue procedures such as eliciting personally meaningful activities, inviting participants to express worries or burdens related to daily life, validating fatigue or non-adherence without blame, and collaboratively selecting feasible home-based activities. Through these structured, value-oriented conversations, participants and staff identified personally meaningful goals and activity plans.

Frailty has been conceptualized as both a physical phenotype and an accumulation of multidimensional deficits, underscoring the importance of early, person-centered preventive strategies in community-dwelling older adults [8,9]. While exercise-based interventions have demonstrated benefits, there remains a gap in understanding how conversational processes, such as value elicitation, emotional acceptance, and shared decision-making, may contribute to sustaining behavior change in preventive care settings.

This practice-based quality improvement (QI) initiative primarily aimed to explore the feasibility of integrating structured autonomy-supportive dialogue into routine daycare services to support voluntary frailty-prevention behaviors among older adults. Secondary exploratory aims included observing practice adaptations during implementation and monitoring trends in physical function over time using the SF-BBS. This project was conducted as a QI activity within routine care rather than as a hypothesis-driven clinical trial; therefore, the quantitative findings were interpreted as exploratory monitoring signals rather than evidence of intervention efficacy.

## Materials and methods

Context

According to the municipal long-term care insurance plan, the number of care workers decreased from 2,605 to 2,260 between 2019 and 2022 [8]. The same public planning document reports that Kitami City had an aging rate of 34.5% as of September 2023 and identifies caregiving workforce development and retention as urgent issues [8]. These data highlight a regional service-delivery challenge involving increasing care demand and workforce constraints.

Access to rehabilitation is limited in the region. In 2021, only six daycare rehabilitation facilities with mandated rehabilitation professionals were available: 7.39 per 10,000 certified older adults, well below the national average of 12.42 [9]. By contrast, most daycare-type facilities (n = 58) operate without rehabilitation professionals, and although functional training instructors are formally required, implementation standards are minimal, and program quality varies widely.

The participating day service primarily served adults in their late 80s to early 90s with mobility limitations related to osteoarthritis, frailty, or chronic pain. Many participants had reduced physical activity due to loss of household or farm-related roles and limited family support, reflecting typical conditions in the region’s semi-rural older population.

The intervention was implemented by a supervising physiotherapist with a background in neurorehabilitation and prior research on motor sense, specifically the sense of agency and ownership, which informed the emphasis on supporting autonomous, self-initiated action consistent with SDT. Because the dialogical process was primarily facilitated by this single physiotherapist, the facilitator’s professional background, communication style, and pre-existing relationship with participants should be understood as part of the implementation context. Potential facilitator-specific influence was therefore considered when interpreting the findings.

Although Japan’s long-term care system emphasizes “self-support” in principle, the prevailing culture in many daycare facilities, including the participating site, remains centered on traditional caregiving tasks such as bathing, meals, and safety monitoring. Service delivery is typically shaped by care managers’ plans, often prioritizing routine assistance over autonomy-supportive practice. Within this context, there was a practical need to strengthen autonomy-supportive care and to promote voluntary daily activity without increasing staff workload or introducing new treatment programs, which directly shaped the design and real-world feasibility of the present QI initiative.

Study design and setting

This project was conducted as a practice-based QI initiative embedded within routine service delivery at a community daycare (day service) center in Kitami City, Hokkaido, Japan. The QI activity was carried out from July 2022 to June 2023 to explore the feasibility of supporting voluntary, home-based activity through structured, autonomy-supportive dialogue between staff and service users. The intervention and evaluations were implemented as part of standard care and administrative practice; quantitative analyses were performed post hoc for monitoring and exploratory learning purposes, rather than for hypothesis-driven evaluation.

Participants and eligibility

**Figure 1:**
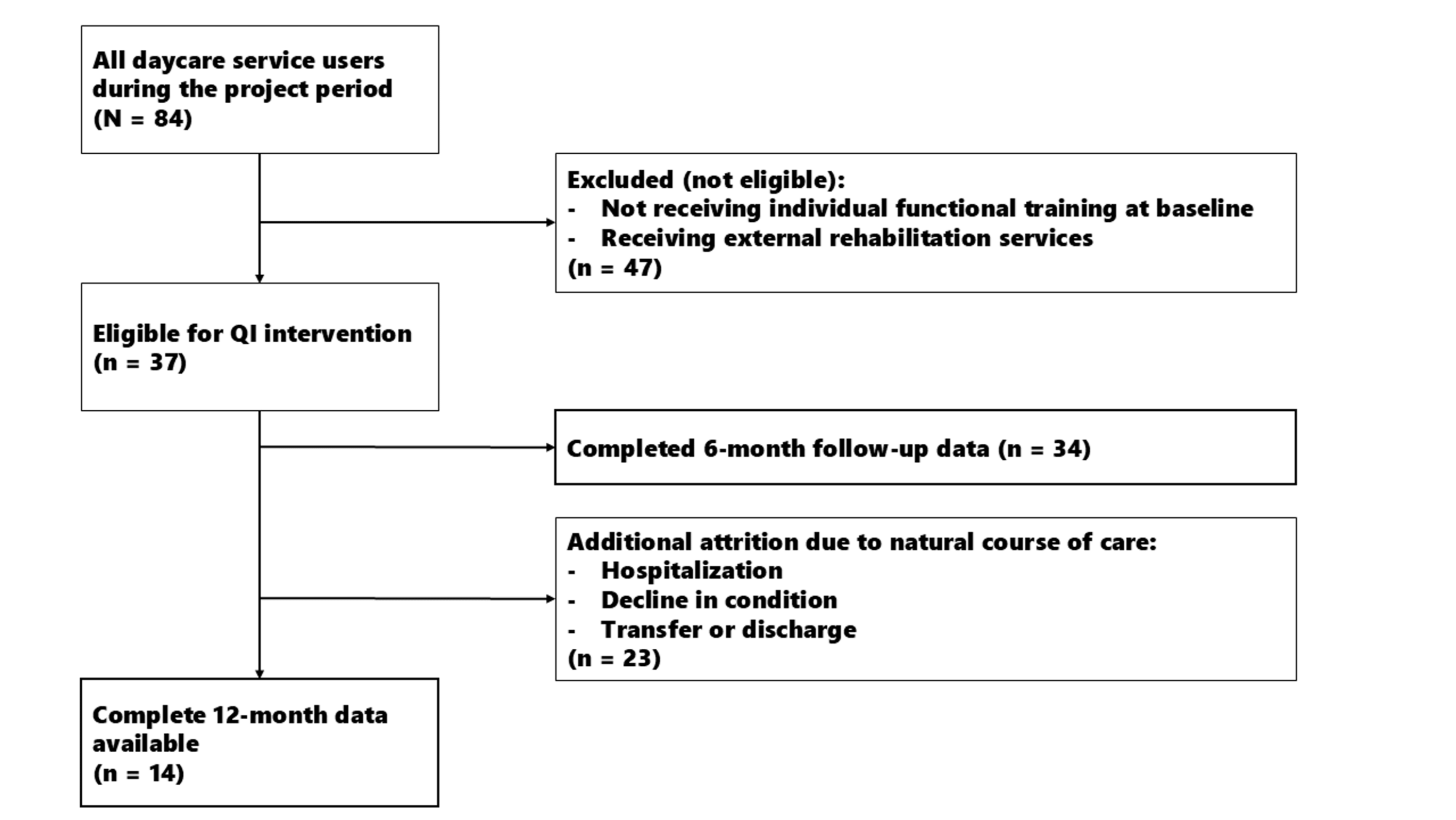
Flow of participant inclusion and natural attrition during the quality improvement project At the start of the project period (June 2023), 84 individuals were enrolled in the daycare center. Of these, 47 either did not participate in individual functional training or were concurrently receiving external rehabilitation services, leaving 37 eligible participants for the quality improvement initiative. Among them, 34 completed the six-month follow-up, and 14 had complete data available at the 12-month assessment. Twenty-three participants discontinued follow-up due to expected changes in their care trajectory, including hospitalization, health deterioration, transfer, or discharge.

Service users who were regularly attending the participating daycare center and receiving routine, individualized functional training at the start of the project were considered for inclusion. Participants were eligible if they (1) were regularly attending the daycare service at baseline and (2) were not receiving structured or ongoing rehabilitation services from external medical institutions. Participants were excluded if they (1) were engaged in physician-directed rehabilitative programs outside the daycare service that could substantially influence functional changes independent of the QI activities or (2) consent or agreement for the anonymized use of routine care data could not be obtained from the participant or, where appropriate, from a family member or proxy through standard facility procedures (Figure 1).

A convenience sample of 37 participants met the eligibility criteria and were enrolled. Because participants were drawn from users already receiving routine individualized functional training at a single daycare center, the sample may have overrepresented individuals who were clinically stable enough to participate in the dialogue process and routine functional monitoring. Basic participant characteristics (age, sex, care/support level, and baseline frequency of attendance) were extracted from service records.

QI intervention: structured dialogical engagement

The QI intervention comprised a semi-structured, four-step dialogical process implemented by a supervising physiotherapist who was not responsible for delivering formal rehabilitation services within the facility, but was routinely involved in QI and functional monitoring. The intervention did not involve the introduction of new structured exercise programs or additional therapeutic modalities; rather, it focused on shaping the content and structure of conversations to support internalization of activity. This approach was not delivered as a formal motivational interviewing or counseling program. Instead, it was a structured, autonomy-supportive dialogue embedded within routine daycare interactions, aimed at linking participants’ personally meaningful values with feasible home-based activities and ongoing functional monitoring.

**Figure 2:**
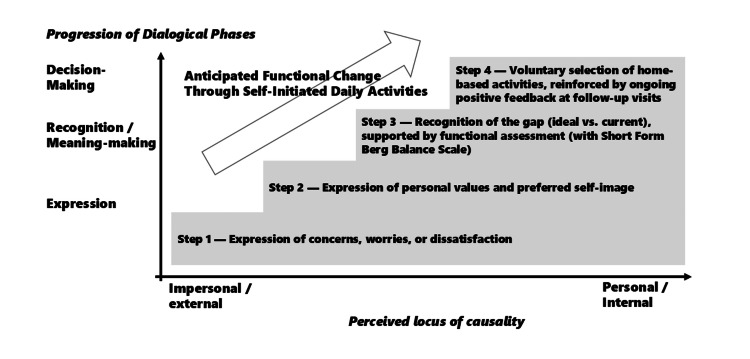
Conceptual framework guiding the dialogical approach This schematic illustrates the working conceptual framework used to guide structured dialogue during the quality improvement initiative. The model integrates principles from self-determination theory (SDT) [5-7] and dialogical practice. It represents a practice-derived heuristic framework and not a tested causal model.

The four steps were as follows: (1) Elicit concerns and constraints - open questions to identify physical complaints, perceived burdens, and anxieties related to daily activities. (2) Elicit values and ideal self - guided prompts to surface personally meaningful activities and the participant’s ideal daily life (e.g., specific social roles, hobbies). (3) Identify gaps - presentation and discussion of current functional status (using measurable indicators) relative to the ideal state; collaborative identification of specific deficits or barriers. (4) Co-develop feasible home-based activities - shared decision-making to agree on simple, safe, and contextually feasible activities to be carried out at home between visits (Figure 2). Biestek’s principles were operationalized through specific conversational behaviors embedded within this four-step process. Individualization was reflected in eliciting each participant’s personally meaningful activities and daily roles. Purposeful expression of feelings was supported through open questions about worries, burdens, and frustrations related to physical decline. Acceptance was reflected in validating fatigue, hesitation, or non-adherence without blame. Self-determination was supported by collaboratively selecting feasible home-based activities rather than prescribing standardized exercises. The fixed elements of the intervention were the four-stage dialogue structure, value clarification, review of agreed home-based activities, and autonomy-supportive feedback. Adaptable elements included the wording of prompts, degree of repetition, pacing of reflection, and adjustment of goals according to cognitive status, fatigue, physical symptoms, daily context, and participant preference. These adaptations were made within routine QI reflection to preserve the core dialogical principles while responding to individual needs.

After the four conversational steps, participants received follow-up at each daycare visit. During these interactions, staff reviewed the participant’s progress with the agreed-upon home activities through open, supportive dialogue. Importantly, positive and autonomy-supportive feedback was provided regardless of the level of adherence. When activities had not been carried out, the physiotherapist explored the participant’s reasons (e.g., fatigue, competing responsibilities), validated these experiences, and emphasized the participant’s ability to self-monitor and make appropriate health-related decisions. This approach was intended to reinforce self-efficacy and to sustain engagement through autonomy-supportive rather than compliance-oriented communication.

All initial structured dialogues were conducted by a single supervising physiotherapist with extensive experience in community-based rehabilitation and routine involvement in interprofessional care planning within the local long-term care system. Other facility staff participated in brief follow-up interactions during routine care, but they did not independently deliver the initial four-step dialogue. Although no specific SDT- or Biestek-focused training program was provided for this project, the facilitator had prior professional familiarity with these frameworks through clinical practice, literature-based learning, and regular collaboration with social workers in community care. Accordingly, the present initiative demonstrates feasibility under a single-facilitator implementation model rather than provider-independent reproducibility. The semi-structured dialogue script and conceptual fidelity checklist may provide a basis for future training of other care staff or rehabilitation professionals, but whether the approach can be delivered with comparable fidelity across providers requires further evaluation.

Primary pragmatic outcome

Physical function was monitored using the Short-Form Berg Balance Scale (SF-BBS) [10] at three routine time points: baseline (at or within two weeks of project initiation), six months, and 12 months. The SF-BBS was administered without modification as part of routine functional monitoring within the facility. Assessments were conducted by the supervising physiotherapist involved in the QI initiative and were not blinded or independent from the intervention process. The original source of the instrument is appropriately cited, and test-retest reliability in older adults has been previously reported [11].

Process and contextual data

Process data included the content summaries of dialogues (summarized into recurring themes and recorded), attendance frequency (visits per week, extracted from service logs), and reasons for missing follow-up (e.g., hospitalization, service discontinuation). All personal identifiers were removed, and data were coded prior to analysis.

Handling of missing data

Due to the QI context, not all participants had complete data at each time point (reasons included hospitalization, service discontinuation, or data recording omissions). The primary analytic approach used complete-case analysis for participants with available values at the relevant time points. Imputation was not attempted because missingness primarily reflected clinically meaningful care-trajectory events, including hospitalization, health deterioration, transfer, or discharge, rather than random data loss. Complete-case analyses were therefore used descriptively, with explicit acknowledgment that participants with longer-term follow-up may represent a relatively more stable subgroup. Sensitivity analyses were performed on larger subsets where appropriate, such as the baseline-to-six-month comparison among participants with data at both time points.

Statistical analysis (exploratory)

Statistical analyses were conducted for exploratory monitoring purposes within the QI context and were not intended as confirmatory hypothesis testing. Descriptive statistics (means ± SD or medians and interquartile ranges, as appropriate) were used to summarize participant characteristics and outcome measures. P-values are reported for transparency only and were interpreted descriptively, with emphasis placed on observed patterns, effect sizes, and clinical plausibility rather than statistical significance. Two complementary exploratory analyses were conducted. First, an available-pair baseline-to-six-month comparison was performed among participants with paired data at these two time points (n = 34). Changes in SF-BBS scores were examined using the Wilcoxon signed-rank test due to the non-normal distribution of change scores. Second, a complete-case repeated-measures analysis was conducted among participants with available observations at baseline, six months, and 12 months (n = 14). Overall differences across the three time points were assessed using the Friedman test. When the overall test indicated a difference across time points, pairwise comparisons were conducted using Wilcoxon signed-rank tests. Effect sizes (r) were calculated for nonparametric comparisons to provide descriptive magnitude estimates. Statistical analyses were performed using R version 4.4.1 in RStudio.

Data management and confidentiality

All project data were recorded on facility forms and entered into a secure, password-protected database by facility staff. Only anonymized, coded data were used for analysis. Access to identifiable data was limited to on-site clinical staff and the QI project lead. Data storage complied with institutional data governance policies.

Ethical considerations

This activity was implemented as a QI project within routine service delivery. In Japan, activities that constitute medical or biological research involving human subjects are generally considered under national ethical guidelines and institutional review procedures. The cooperating institution’s ethics office reviewed the project documentation and determined that this activity, conducted as a QI initiative within routine service delivery, did not require formal ethical review (notification letter attached). No additional procedures, assessments, or risks were introduced beyond routine service monitoring. Nevertheless, the project followed ethical principles consistent with the Declaration of Helsinki and relevant national guidelines. Oral and written consent for routine functional assessment and anonymized use of service data for QI purposes was obtained from all participants, or from family members or proxies where applicable, through standard facility procedures. For participants with cognitive impairment, explanations were provided to the participants whenever possible and to family members or proxies as appropriate at the time of service and functional training agreement. Participants were included only when consent or agreement for the use of routine care data was confirmed through these standard procedures. The ethics notification letter and consent materials are available upon request.

Reporting standards and checklist

This manuscript follows the SQUIRE 2.0 (Standards for Quality Improvement Reporting Excellence) reporting framework as a guiding reference, where applicable, and statistical reporting conforms to conventional practice for exploratory QI projects [12]. Supporting materials, including the semi-structured dialog script and a conceptual intervention fidelity checklist (Appendices A and B), are provided to enhance transparency and reproducibility.

Fidelity to the guiding frameworks was monitored reflectively using a conceptual checklist rather than through formal adherence scoring or external auditing. In addition to reflective review, observable adherence indicators were used to support consistency, including whether the four dialogue stages were completed, whether personally meaningful goals were documented, whether agreed home-based activities were recorded, and whether follow-up conversations reviewed those activities using autonomy-supportive feedback. These elements were reviewed as part of routine QI reflection to support consistency across interactions while allowing contextual adaptation.

## Results

Participant characteristics

A total of 37 participants met the eligibility criteria and were included in the QI initiative. The mean age was 88.7 ± 5.6 years, and 78.4% were female. The participants attended the daycare center an average of 2.0 ± 1.0 days per week at baseline. The distribution of care/support levels and baseline attendance frequency is summarized in Table 1. Of these, 34 participants had complete six-month follow-up data, and 14 had complete 12-month data (Table 1). Attrition was primarily due to hospitalization, changes in care needs, or transfer/discharge, typical natural variations in a long-term care population, rather than withdrawal related to the QI activities.

**Table 1:** Baseline characteristics of participants included in the quality-improvement initiative Baseline demographic and service-use characteristics of participants enrolled in the quality-improvement initiative (n = 37). Variables include age, sex, care/support level under the Japanese long-term care insurance system, and baseline frequency of daycare attendance. Follow-up sample sizes at six months (n = 34) and 12 months (n = 14) reflect natural attrition due to changes in health status or care needs, rather than withdrawal related to the quality-improvement activities.

		Eligible for QI intervention (37)	Completed six-month follow-up (34)	Complete 12-month follow-up (14)
Age (mean ± SD)		88.7 ± 5.5	88.4 ± 5.6	89.4 ± 5.5
% female		78.4	76.5	78.6
Attendance at the daycare center, on average, days per week (mean ± SD)		2.0 ± 1.0	2.0 ± 1.0	1.9 ± 0.8
Distribution of care/support level, n (%)	Community-based care	1 (2.7)	0 (0.0)	0 (0.0)
	Support level 1	9 (24.3)	9 (26.5)	2 (14.3)
	Support level 2	7 (18.9)	7 (20.6)	2 (14.3)
	Care level 1	11 (29.7)	9 (26.5)	6 (42.9)
	Care level 2	8 (21.6)	8 (23.5)	4 (28.6)
	Care level 3	1 (2.7)	1 (2.9)	0 (0.0)
	Care level 4	0 (0.0)	0 (0.0)	0 (0.0)
	Care level 5	0 (0.0)	0 (0.0)	0 (0.0)

Fidelity of the dialog characteristics

The semi-structured dialogical method was implemented consistently according to the predefined four-stage structure, as monitored through reflective review using the conceptual fidelity checklist. Conversations progressed through four predefined stages: (1) expression of physical concerns, (2) clarification of personal values and desired lifestyle, (3) identification of the gap between current function and desired state, supported by SF-BBS results, and (4) collaborative selection of home-based activities. Representative conversational themes and participant quotes are summarized in Table 2. These excerpts were selected to illustrate recurring themes observed across the dialogue summaries, including physical concerns, personally meaningful values, recognition of functional gaps, and collaboratively selected home-based activities. They are presented as descriptive examples of the implementation process rather than as formal qualitative evidence. Practice-based adaptations were not considered protocol deviations but were treated as expected iterative refinements consistent with QI practice. The complete version of Table 2 summarizing dialogical themes for all participants is presented in Appendix C.

**Table 2:** Representative dialogical themes and illustrative participant statements across the four-stage process This table presents representative excerpts selected to illustrate recurring themes observed across the dialogue summaries during the four-stage dialogical process. The examples are intended to demonstrate how the dialogue structure was implemented in practice and are not presented as formal qualitative analytic findings. The complete participant-level summary (n = 37) is provided in Appendix B. Care/support level corresponds to the Japanese long-term care insurance system, and Independence (Physical/Cognitive) refers to the Japanese national functional independence classification used in long-term care settings.

ID	Visits/week	Care level	Independence (physical/cognitive)	Main health concerns	Expressed values and ideal self	Perceived barriers/deficits	Agreed-on home-based activities
Subj 1	1	Support level 1	J1 / I	Knee pain and fear of going out by bus	Wishes to resume social dancing and outdoor activities	Needs posture control and lower limb strength for pain management	Sit-to-stand training, tandem stance, short outdoor trips using taxi assistance
Subj 2	2	Care level 2	A2 / II a	Visual impairment, walking with wall support	Hopes to live independently without increasing family support	Requires lower-limb strength for dynamic balance	Sit-to-stand and tiptoe exercises; use of a pedal exerciser at home
Subj 3	2	Support level 2	J1 / Independent	Rheumatoid arthritis, limited ROM, and THA	Wishes to go out safely to cafés or the library	Needs hip abductor strength and single-leg stability	Hip abduction exercises in lying/standing; light household tasks
Subj 37	1	Community-based care	J1 / Independent	Knee and back pain make gardening difficult	Wants to maintain her flowerbed as her strength declines	Leg strength and postural management during gardening	Sit-to-stand and toe-raise; install an outdoor chair for gardening tasks

Intervention adaptation during implementation

Although the intervention was guided by a semi-structured dialogue script, several practice-based adaptations were noted during routine implementation. These adaptations were not systematically evaluated as separate outcomes but were recorded as part of routine QI reflection. For participants with cognitive impairment, staff used repeated and simplified reflection questions and more frequent anchoring to personally meaningful activities. For example, when watering flowers was identified as a valued activity, staff intentionally revisited this theme during follow-up conversations (“How are your flowers today?”) to support recall of prior successful actions and continuity of engagement. For participants whose outdoor activity increased, conversational content was adjusted to address topics such as pacing, fatigue management, and fall-risk awareness as these issues emerged in routine interactions. Daily follow-up occurred whenever participants attended the center. Staff confirmed activity engagement through open conversation and provided autonomy-supportive feedback even when participants did not complete planned activities, for example, by acknowledging fatigue as appropriate self-management rather than non-adherence. These observations are presented as descriptive examples of QI flexibility, in which conversational processes were adapted to individual needs while preserving the overarching intervention principles.

Intended outcomes

The intended outcomes of the initiative were increased engagement in autonomous, self-directed physical activity, greater participation in meaningful home-based exercise, and associated functional changes measured using the SF-BBS. During routine follow-up conversations, participants described newly developed routines such as daily walking or purposeful household activity, and some expressed increased confidence in managing their functional health. These observations were not formally analyzed as qualitative outcomes and should be interpreted descriptively.

Unintended outcomes

Several positive, non-planned outcomes were noted during routine monitoring. These observations were not systematically measured and should be interpreted descriptively. They included increased peer-to-peer conversation, with participants spontaneously sharing their activity efforts with others; more autonomous use of the training area, reducing the need for staff prompting; greater specificity in functional training plans, enhancing communication between daycare staff and care managers; and gradual diffusion of autonomy-supportive practices within the facility, with the number of participants requesting functional training increasing from 10 in 2022 to 28 in 2023. These counts reflect facility-level administrative records and were not derived from a controlled comparison. Adverse events were monitored through routine facility incident-reporting procedures and staff observation during ordinary service delivery. No adverse events attributable to the dialogue process were identified through these routine monitoring procedures.

Exploratory quantitative changes in physical function

**Figure 3:**
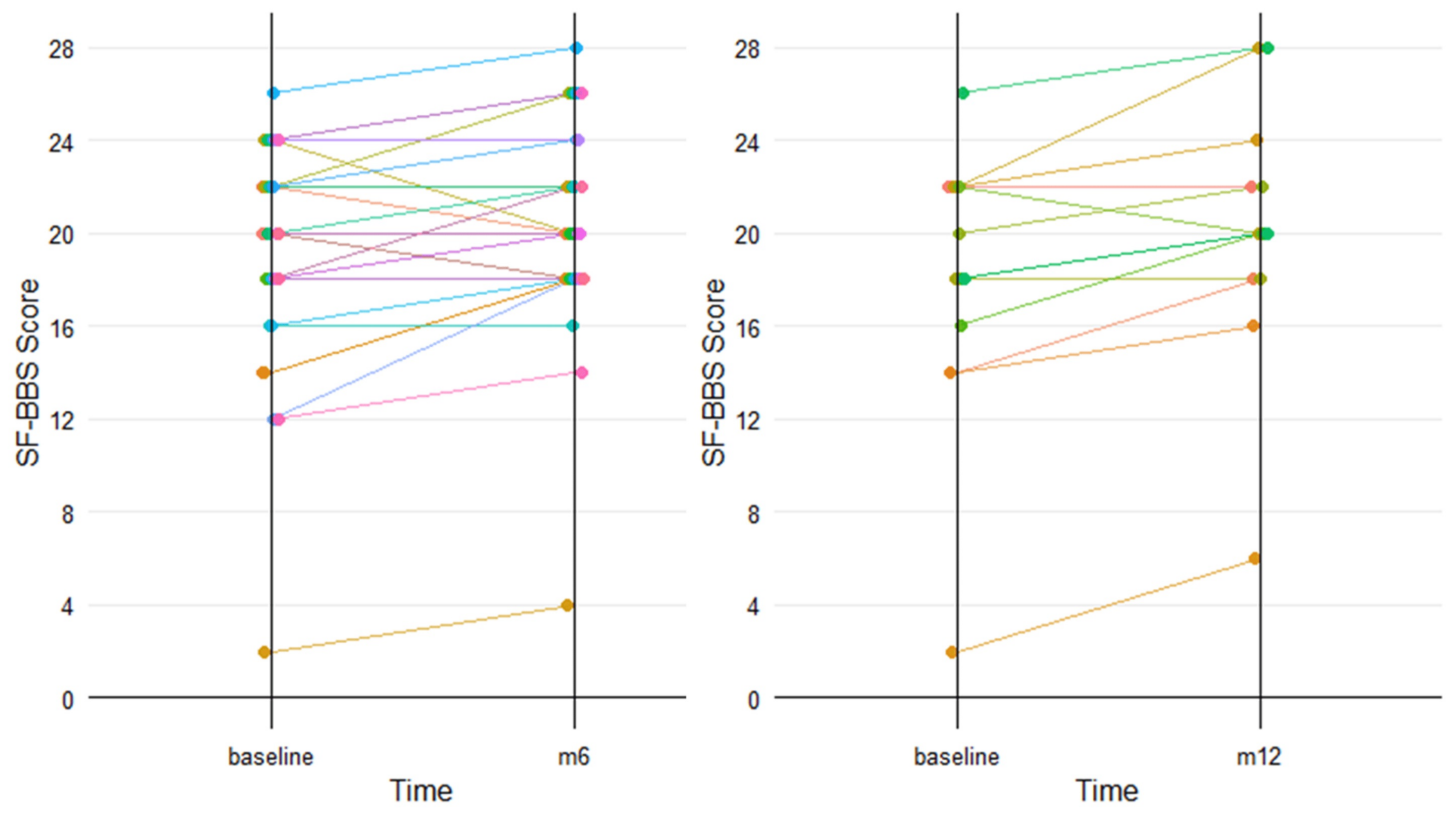
Individual trajectories of Short-Form Berg Balance Scale (SF-BBS) scores during the quality-improvement initiative Each point represents an individual participant, with paired observations shown between baseline and follow-up assessments. The left panel illustrates changes from baseline to six months among participants with available data (n = 34), and the right panel illustrates changes from baseline to 12 months among those with longer-term follow-up (n = 14). Lines connect repeated measurements from the same individual to visualize within-person trajectories over time. These scatterplots are presented to highlight individual-level variability and patterns of engagement in a real-world quality-improvement context, rather than to imply causal effects of the intervention.

These analyses were exploratory and intended to complement, rather than replace, the practice-based findings of this QI initiative. To describe functional trends over time, two complementary exploratory analyses were performed on SF-BBS scores. First, among participants with available paired observations from baseline to six months (n = 34), SF-BBS scores showed a median increase of +2 points (IQR: 0.0-2.0). Exploratory analysis indicated an observed positive shift in scores (Wilcoxon signed-rank test: Z = −2.83, p < 0.01, |r| = 0.59). Second, among participants with complete three-timepoint data at baseline, six months, and 12 months (n = 14), SF-BBS scores showed a positive trend over time. Pairwise exploratory comparisons within this complete-case subset are presented in Table 3, and the overall three-timepoint comparison is presented in Table 4. The negative Z values reflect the direction of signed ranks in the Wilcoxon test and do not indicate functional decline. Effect sizes are therefore reported as absolute values to describe the magnitude of observed change. These p-values are reported for transparency and should not be interpreted as confirmatory evidence of intervention efficacy. Given natural attrition, complete-case analysis, and the absence of a control group, these findings should be interpreted as descriptive functional trends within a real-world QI context. Scatterplots illustrating individual change trajectories are presented in Figure 3. These figures are intended to visualize individual-level variability and engagement patterns rather than to imply causal effects. Additional exploratory statistical summaries, including complete-case pairwise and overall three-timepoint comparisons, are provided in Tables 3-4.

**Table 3:** Exploratory pairwise comparisons among participants with complete three-timepoint data (n = 14) Pairwise comparisons among the complete-case subset are presented in Table 3. Analyses were restricted to participants with available observations at baseline, six months, and 12 months. These comparisons are separate from the available-pair baseline-to-six-month analysis among all participants with paired data (n = 34), which is reported in the text. P-values are reported for transparency and should be interpreted descriptively rather than as confirmatory evidence of intervention efficacy. Negative Z values reflect the direction of signed ranks in the Wilcoxon test and do not indicate functional decline. Effect sizes are presented as absolute values to indicate the magnitude of observed change. No causal inference is implied.

Comparison	n	Test	Statistic	p-value	Z	Effect size (|r|)
Baseline versus six months	14	Wilcoxon signed-rank	V = 4	0.01754151	-2.620863	0.700455
Baseline versus 12 months	14	Wilcoxon signed-rank	V = 4.5	0.01147875	-2.7623112	0.7382587

**Table 4:** Exploratory overall comparison of Short-Form Berg Balance Scale scores across three time points using the Friedman test (n = 14) Overall three-timepoint comparisons among the complete-case subset are presented in Table 4. Analyses were restricted to participants with available observations at baseline, six months, and 12 months. P-values are reported for transparency and should be interpreted descriptively rather than as confirmatory evidence of intervention efficacy. No causal inference is implied.

Overall comparison	n	Test	χ²(df)	p-value
Baseline / 6 mo / 12 mo	14	Friedman	χ² = 11.87 (2)	0.002646

## Discussion

Interpretation of findings

This QI initiative suggests that structured, autonomy-supportive dialogue can serve as a feasible entry point for fostering voluntary engagement in preventive activities within routine daycare settings. Rather than introducing new programs, the approach relied on reconfiguring everyday interactions, indicating that meaningful shifts may emerge from dialogical reframing of existing practices.

Several contextual factors likely contributed to these developments. Although the facility did not initially operate with an explicit autonomy-supportive culture, staff were experienced in relationship-based caregiving and became receptive to dialogical practices as positive user responses accumulated. The physiotherapist’s background in community rehabilitation provided a coherent framework for translating participants’ narratives into individualized activity plans. Participants also demonstrated openness to ongoing reflective conversations, enabling meaningful and achievable goals to be co-created through dialogue.

Taken together, these observations suggest that even in traditionally structured daycare environments, dialogical engagement may serve as a practical entry point for shifting from prescriptive routines toward more autonomy-supportive, individualized care. Although causal inference is not warranted, descriptive trends indicated score shifts even within a cohort of very advanced age (mean 88.7 years), a population in which functional decline is commonly reported in longitudinal observations. This contextual factor underscores the need for cautious but attentive interpretation of observed changes.

Transferability and generalizability

Many essential components of the dialogue, i.e., value clarification, reflective questioning, positive reinforcement, and iterative goal-setting, can be performed by trained care staff and do not necessarily require specialist-level expertise. This suggests that the approach may be transferable to other daycare facilities or community care contexts.

However, integrating functional assessment (e.g., interpreting SF-BBS scores) into the dialogue may require either the presence or periodic supervision of rehabilitation professionals, particularly when participants express concerns related to mobility or fall risk. Facilities in urban settings, where access to such professionals is more readily available, may therefore find implementation easier, in rural regions with limited rehabilitation resources, hybrid models-such as periodic consultation, periodic remote consultation by rehabilitation professionals (e.g., tele-rehabilitation), or structured training-may support broader application. Implementation may also depend on organizational readiness and leadership support, which are important contextual determinants in QI initiatives.

To enhance reproducibility, future implementation could standardize the dialogical elements into brief structured prompts and reflection checklists usable by non-specialist staff. The current report reflects an early implementation phase rather than a finalized protocol, and iterative refinement is ongoing.

Comparison with the literature

The findings are broadly consistent with previous evidence suggesting that SDT-based interventions can support autonomous motivation and sustained engagement in health-related behaviors [13,14]. In the present QI initiative, participant statements recorded during routine dialogue appeared to include expressions moving from externally prompted activity toward more personally meaningful reasons for activity. However, because no formal qualitative methodology or validated motivational assessment was applied, these observations should be interpreted as descriptive practice-based signals rather than evidence of confirmed motivational change.

Additionally, the relational characteristics of the dialogue were consistent with Biestek’s foundational social work principles, including respect for individuality, acceptance, and support for self-determination [7]. These principles may have helped create a conversational environment in which participants could express concerns, values, and preferences more openly than in prescriptive care interactions. However, this interpretation remains exploratory.

The culturally embedded notion of nattoku may provide a useful interpretive lens for understanding how participants made sense of activity recommendations. In Japanese care contexts, nattoku refers not simply to cognitive agreement but to a personally meaningful sense of acceptance that integrates understanding, emotion, and relational trust [15]. In this study, nattoku is used as an interpretive concept rather than as an empirically measured construct. While culturally grounded, similar relational processes have been discussed in international literature in terms of cognitive-emotional coherence, shared decision-making, or therapeutic alliance. Thus, although terminology differs, the underlying relational dynamics may have broader relevance.

From a broader public health perspective, the initiative aligns with the WHO “Rehabilitation 2030” framework [16], which emphasizes person-centeredness, community-based implementation, and support for functional participation rather than a narrow focus on impairment. The dialogical method may offer one practical approach for operationalizing these principles in everyday care, especially in resource-limited settings, although further evaluation using formal qualitative and mixed-methods designs is needed.

Limitations

This QI initiative was not designed to establish causal inference. Attrition over 12 months was primarily related to hospitalization, health deterioration, or care transitions, resulting in a longer-term follow-up sample that may represent a relatively more stable subgroup. This survivorship pattern may have influenced observed functional trends, and effect sizes calculated within the complete-case subset may overestimate true population-level effects.

The use of a convenience sample may also have introduced selection bias. Participants who were already engaged in individualized functional training and who completed at least one full dialogue cycle may have been more clinically stable, more receptive to interaction, or more motivated than other daycare users. Therefore, the findings may not represent all users of daycare services.

Because the dialogical process was primarily facilitated by a single supervising physiotherapist, the extent to which findings reflect the structured method itself versus facilitator-specific relational style cannot be determined. This single-facilitator structure may have influenced participant engagement, communication patterns, and observed outcomes. Inter-provider applicability was not evaluated; future studies should examine whether trained care staff or rehabilitation professionals can deliver the approach with comparable fidelity, ideally using standardized training, independent fidelity assessment, and inter-rater reliability procedures.

Quantitative changes in SF-BBS scores may also reflect natural variation, regression to the mean, assessment bias, or contextual influences unrelated to the dialogical process. In addition, SF-BBS assessments were not blinded or independent from the intervention process. Therefore, findings should be interpreted as descriptive signals within a real-world implementation context rather than evidence of intervention efficacy.

Measurement limitations should also be acknowledged. Comprehensive frailty indicators-such as grip strength, gait speed, nutritional assessment, and cognitive testing-were not systematically collected. As a result, interpretation relies primarily on SF-BBS scores and participant narratives, limiting multidimensional assessment of frailty [17,18]. Although the dialogical process generated rich qualitative material, no formal qualitative analytic method (e.g., thematic analysis or conversation analysis) was applied, constraining deeper examination of how participants interpreted and responded to the dialogue process [19,20]. Accordingly, conversational observations should be interpreted as descriptive examples of implementation processes rather than as formal qualitative findings. Fidelity monitoring was reflective and practice-based rather than objectively scored; no external audit, inter-rater reliability assessment, or formal adherence rating was conducted.

Finally, the initiative was conducted in a single facility with a specific organizational culture and staffing structure. Generalizability may therefore be limited, and replication across diverse settings will be necessary. As with many real-world QI initiatives, the intervention evolved incrementally during implementation. The four-stage dialogue structure and autonomy-supportive feedback remained fixed, whereas prompt wording, repetition, pacing, and goal adjustment were adapted according to participant needs and context. This flexibility supported contextual fit but may reduce procedural reproducibility.

Implications for practice

Japan’s long-term care system formally emphasizes self-support and autonomy, yet practical models for translating these principles into everyday interactions remain limited. This initiative suggests that autonomy-supportive dialogue can be embedded within routine care through modest reframing of existing professional practices, without introducing new programs, staffing, or equipment. Rather than restructuring services, the approach involved restructuring conversations.

From an implementation perspective, training for care staff, periodic supervision by rehabilitation professionals, and iterative refinement of conversational techniques may support greater consistency. In resource-constrained settings, dialogical engagement may represent a pragmatic pathway toward strengthening motivation and participation while aligning preventive care with routine service delivery.

## Conclusions

This QI initiative demonstrates the practical feasibility of embedding autonomy-supportive dialogue within routine geriatric daycare services. Although causal effects were not established, the findings suggest that dialogical reframing of everyday interactions may contribute to voluntary engagement in preventive care. Future studies should examine this approach across diverse care settings using controlled comparative designs, independent outcome assessment, and mixed-methods evaluation to clarify both implementation processes and participant outcomes.

## Figures and Tables

**Figure 4:**
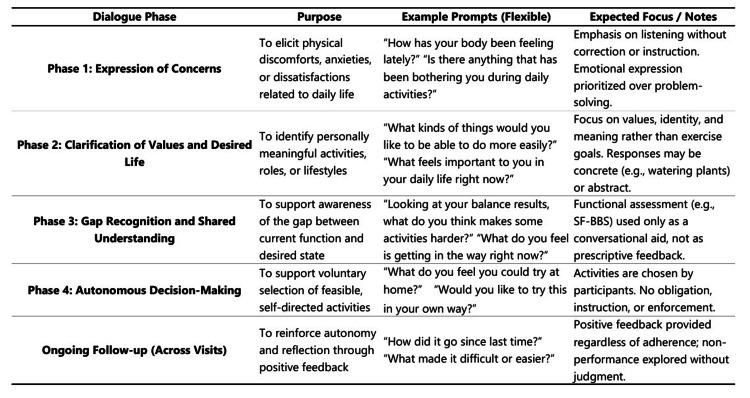
Semi-structured dialog guide used in the quality improvement (QI) initiative This guide served as a flexible conversational framework rather than a prescriptive protocol. Wording and depth were adapted according to participant cognition, values, and daily context. Adaptation for cognitive impairment: For participants with cognitive impairment, previously identified values were reinforced using repeated, concrete prompts to support recall and continuity of meaning. Role of the facilitator: Dialogues were facilitated by a supervising physiotherapist with experience in community rehabilitation and autonomy-supportive communication. No additional formal training specific to this project was conducted.

**Figure 5:**
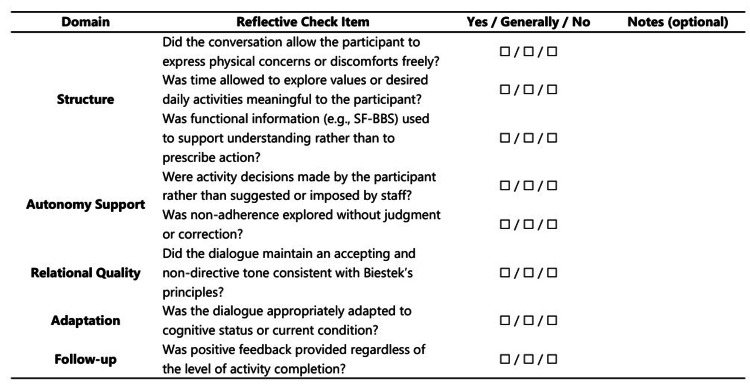
Conceptual intervention fidelity checklist This reflective checklist was used to monitor alignment with autonomy-supportive dialogical principles. It was not employed for scoring, enforcement, or performance evaluation. Use in practice: The checklist supported reflective consistency rather than procedural compliance and was not linked to individual outcome measurement. Handling of variability: Variations in wording, duration, and conversational depth were considered appropriate provided that core autonomy-supportive intent was preserved.

## Appendices

Supplementary materials include (1) the semi-structured dialog guide used during implementation; (2) a conceptual intervention fidelity checklist; (3) the complete version of Table 2 summarizing dialogical themes for all participants. These materials are provided to enhance transparency, reproducibility, and contextual clarity. An official written notification from the institutional ethics office confirming that formal ethical review was not required is available upon request.

Appendix A: semi-structured dialog guide used during the implementation

Appendix B: conceptual intervention fidelity checklist

Appendix C: complete version of Table 2 summarizing dialogical themes for all participants

**Table 5:** Complete participant-level summary of dialogical themes and agreed home-based activities (n = 37) This table provides the full participant-level summary corresponding to the representative excerpts presented in Table 2. All 37 eligible participants are included. Information reflects dialogical themes identified during the four-stage conversational process, including expressed values, perceived barriers, and collaboratively selected home-based activities. Data are anonymized and presented to enhance transparency and contextual understanding of the intervention process.

ID	Visits/week	Care Level	Independence (Physical / Cognitive)	Main Health Concerns	Expressed Values and Ideal Self	Perceived Barriers / Deficits	Agreed Home-based Activities
Subj 1	1	Support Level 1	J1 / I	Knee pain and fear of going out by bus	Wishes to resume social dancing and outdoor activities	Needs posture control and lower limb strength for pain management	Sit-to-stand training, tandem stance, short outdoor trips using taxi assistance
Subj 2	2	Care Level 2	A2 / II a	Visual impairment, walking with wall support	Hopes to live independently without increasing family support	Requires lower-limb strength for dynamic balance	Sit-to-stand and tiptoe exercises; use of pedal exerciser at home
Subj 3	2	Support Level 2	J1 / Independent	Rheumatoid arthritis, limited ROM, and THA	Wishes to go out safely to cafés or library	Needs hip abductor strength and single-leg stability	Hip abduction exercises in lying/standing; light household tasks
Subj 4	2	Care Level 1	A1 / I	Knee pain, poor standing balance	Hopes to walk short distances without walker in facility	Requires hip muscle strength for posture stabilization	Sit-to-stand with towel squeeze; hallway walking using handrails
Subj 5	2	Care Level 2	A1 / I	Limited ROM of hip/knee, fear of falling	Wishes to maintain independence in simple household tasks	Needs global muscle strength within limited ROM	Mini-squat and side-step; hanging laundry independently
Subj 6	3	Care Level 2	J2 / II a	Experiences shortness of breath and palpitations when moving too much	Wishes to live at home until reaching 100 years old in the next few years	Needs awareness of overall physical condition and appropriate pacing of activity	Gentle sit-to-stand exercise; rest management according to heart rate and breathing condition
Subj 7	2	Support Level 2	A1 / II b	Fatigue during activity due to heart failure and post-valve replacement	Wishes to continue living at home and keep walking as a hobby as long as possible	Enhancement of cardiopulmonary function through calf muscle pump activity	Slow toe-raising exercises; rest management according to heart rate and breathing condition
Subj 8	2	Care Level 2	A1 / II a	Limited range of motion and pain after hip and knee replacement surgeries	Wishes to continue her role in the osteoarthritis patient support group	Improvement of knee joint range of motion and stability based on postoperative progress	Knee control and step exercises; resume neighborhood social gatherings
Subj 9	2	Care Level 1	J2 / I	Knee pain limits walking distance and causes concern for the future	Wishes to continue living at home and chatting outdoors with friends without falling	Needs control of knee joints and strengthening of periarticular muscles	Knee control exercises; resume short outdoor walks using a walker
Subj 10	1	Support Level 1	J2 / I	Back pain makes prolonged housework difficult without walker or handrail	Wishes to live alone as long as possible and feel less fatigued	Strengthening of gluteal and posterior leg muscles	Supine hip lift and sit-to-stand training; go shopping with son using a cart
Subj 11	2	Care Level 1	A1 / I	Feels unstable when walking and falls while gardening; scolded by family	Wishes to walk safely and enjoy gardening without being scolded	Needs lower-limb strength to maintain balance	Slow sit-to-stand and tiptoe exercises; go out to garden using a cane (with family approval)
Subj 12	1	Support Level 1	J1 / Independent	Easily fatigued after myocardial infarction; reluctant to walk or garden	Wishes to maintain health and continue living independently at home	Enhancement of cardiopulmonary function through calf muscle pump activity	Gentle sit-to-stand and toe-raise; resume walking even for short distances
Subj 13	4	Care Level 2	J2 / II b	Knee pain makes sitting and standing difficult	Hopes to walk comfortably and continue annual trip to meet grandchildren	Lower-limb muscle strength and maintenance of home activity level	Slow sit-to-stand and tiptoe exercises; participate in pet care tasks
Subj 14	1	Support Level 1	A1 / I	Back pain after lumbar fracture causes stooped posture and easy fatigue	Wishes to correct posture and maintain stamina to continue shopping and embroidery	Strengthening of back and lower-limb muscles	Pain-free hip lift in supine position; side-lying leg extension; chair adjustment and gradual resumption of seated activities
Subj 15	1	Support Level 1	J2 / I	Knee pain makes it hard to walk without keeping legs extended; decreased walking distance	Wishes to improve gait to continue living with family without burdening them	Knee control and strengthening of periarticular muscles	Knee control exercises in sitting or lying position; use walker and extend walking distance gradually
Subj 16	1	Care Level 1	A1 / I	Low back pain reduces motivation to help with housework	Believes that standing at the kitchen will be easier once back pain improves	Strengthening of posterior thigh muscles	Slow sit-to-stand and tiptoe exercises; place a chair near sink to extend housework time
Subj 17	2	Care Level 1	J1 / I	Knee pain after Achilles tendon surgery makes stair climbing difficult	Wishes to perform household chores and water plants on the second floor daily	Ankle extensor and knee joint muscle strength	Sit-to-stand and tiptoe exercises; mark calendar on days she waters plants
Subj 18	1	Support Level 2	J1 / I	Feels unstable and trips easily when working in the garden	Wishes to safely continue self-sufficient farming lifestyle	Strengthening of posterior leg muscles	Sit-to-stand and tiptoe exercises; use a container as a seat in the garden for safety
Subj 19	5	Care Level 3	A1 / III b	Feels wobbly and unsafe when walking	Wishes to maintain health to avoid burdening family, as bedroom is upstairs	Maintenance of overall body strength	Sit-to-stand and tiptoe exercises; mindful eating with family support
Subj 20	2	Care Level 1	J2 / II b	Recently developed severe back pain, with difficulty raising arms when pain worsens	Wishes to continue weeding and keeping the surroundings of her home clean	Strength of posterior chain muscles from trunk to gluteal region	Combination of seated arm-raise and sit-to-stand exercises; practice of upper-limb movement and mobility during crouched weeding posture
Subj 21	3	Care Level 2	A2 / II a	Knee deformity and upper-limb injury causing frequent falls even with cane use	Wishes to move safely and walk smoothly to avoid burdening family	Strength of hip adductor and surrounding muscles	Combination of hip adduction and sit-to-stand exercises; practice of walking without excessive forward trunk leaning while holding onto furniture
Subj 22	2	Care Level 1	A1 / II b	Bilateral knee pain making transfers and mobility difficult	Wishes to continue housework for her husband and son	Understanding and management of pain-inducing posture; control of foot and ankle muscles	Sit-to-stand and tiptoe exercises in pain-free posture; modification of cleaning tools to avoid excessive bending
Subj 23	2	Support Level 2	J1 / I	Osteoarthritis of the hip making outings increasingly difficult	Wishes to continue social interactions and independent visits to medical facilities	Strengthening of hip muscles	Hip internal and external rotation exercises in pain-free posture; awareness of using the unaffected side during outings
Subj 24	4	Care Level 1	J2 / II b	Feels unmotivated and fears physical decline due to inactivity	Wishes to move freely again like when practicing dance	Decreased activity in daily life	Tandem walking practice using handrails; walk to nearby stores or salon during outings
Subj 25	2	Care Level 1	J1 / Independent	Knee pain and severe diabetes; lives alone and worries about declining physical condition	Wishes to walk uphill to the bus stop by herself	Strength of lower leg muscles	Sit-to-stand and tiptoe exercises; neighborhood walking practice
Subj 26	1	Support Level 1	J1 / I	Cervical and lumbar spondylosis and hip arthroplasty causing daily back pain	Wishes to live without relying on a massage chair	Flexibility of posterior body muscles	Upper-limb elevation and active stretching; marking days when massage chair use was avoided
Subj 27	1	Support Level 1	Independent / I	Chronic renal failure and lumbar spinal stenosis causing leg weakness and fatigue	Lives alone and wishes to walk safely to maintain ability to attend medical appointments	Strength of lower leg muscles	Sit-to-stand and tiptoe exercises; activity management based on fatigue, heart rate, and breathing
Subj 28	2	Support Level 2	J1 / I	Hospitalized previously due to disuse syndrome and wishes to avoid recurrence	Lives alone with distant relatives; wishes to maintain fall-free daily life	Whole-body strength and self-management of diet and hydration	Hip-lift and tandem standing exercises; keeping checklists for diet and fluid intake
Subj 29	1	Support Level 1	J1 / II a	Despite attending a gym, feels physical weakness; experiences dizziness due to Ménière’s disease	Wishes to stay active and walk outdoors without succumbing to aging	Lower-limb strength and safety awareness considering dizziness	Sit-to-stand and tiptoe exercises; introduction of Nordic poles and resumption of gardening activities
Subj 30	1	Care Level 1	A1 / II b	Lumbar spinal stenosis causing back pain and difficulty sustaining manual tasks	Wishes to assist his son in the family construction business	Understanding of condition and posture management	Stretching of hip and thigh muscles; modification of working posture and chair height
Subj 31	2	Care Level 2	A1 / I	Fell outdoors; knee and back pain make long-distance walking difficult	Wishes to continue attending senior club and socializing with friends	Strength of posterior leg muscles and control of trunk forward-leaning posture during walking	Hip-lift exercises; continuation of outdoor walking using two canes
Subj 32	2	Support Level 2	A1 / I	Stooped posture and back pain make household tasks difficult	Wishes to continue independent living by making housework easier	Strength of hip muscles	Hip-lift exercises; pacing household chores with rest breaks to extend activity duration
Subj 33	2	Support Level 2	J1 / Independent	Moved in with family but feels physically and mentally weaker due to inactivity	Wishes to stay able to walk to the convenience store for lunch during the day	Lower-leg muscle strength and foot instability	Toe-raise and tandem walking exercises; taking a slightly longer route during daily walks for lunch
Subj 34	3	Care Level 2	B1 / II a	Knee pain makes walking difficult and feels guilty about burdening her son	Wishes to walk safely indoors and to the hospital using a walker	Posterior leg muscle strength and control of trunk forward-leaning during walking	Toe-raise exercises at dining table; mindful posture during walker or supported walking
Subj 35	4	Care Level 1	J2 / III b	Worsening knee pain leading to shortened step length when walking	Wishes to maintain safe, independent walking indoors despite spending much time alone	Knee joint muscle strength and postural instability	Hip-lift and knee-control exercises; use of handrails to avoid excessive joint loading
Subj 36	1	Support Level 1	A1 / II a	Fatigues easily and has knee pain that interferes with gardening	Wishes to continue helping with household farming and gardening while staying active	Strength of posterior leg muscles and control of trunk forward-leaning during walking	Sit-to-stand and tiptoe exercises; practice of upper-limb movement and mobility during crouched weeding posture
Subj 37	1	Community-based care	J1 / Independent	Knee and back pain make gardening difficult	Wants to maintain her flowerbed as her strength declines	Leg strength and postural management during gardening	Sit-to-stand and toe-raise; install outdoor chair for gardening tasks
